# Automatic Classification of Adventitious Respiratory Sounds: A (Un)Solved Problem? †

**DOI:** 10.3390/s21010057

**Published:** 2020-12-24

**Authors:** Bruno Machado Rocha, Diogo Pessoa, Alda Marques, Paulo Carvalho, Rui Pedro Paiva

**Affiliations:** 1 University of Coimbra, Centre for Informatics and Systems of the University of Coimbra, Department of Informatics Engineering, 3030-290 Coimbra, Portugal; bmrocha@dei.uc.pt (B.M.R.); dpessoa@dei.uc.pt (D.P.); carvalho@dei.uc.pt (P.C.); 2 Lab3R—Respiratory Research and Rehabilitation Laboratory, School of Health Sciences (ESSUA), University of Aveiro, 3810-193 Aveiro, Portugal; amarques@ua.pt; 3 Institute of Biomedicine (iBiMED), University of Aveiro, 3810-193 Aveiro, Portugal

**Keywords:** adventitious respiratory sounds, experimental design, machine learning

## Abstract

(1) Background: Patients with respiratory conditions typically exhibit adventitious respiratory sounds (ARS), such as wheezes and crackles. ARS events have variable duration. In this work we studied the influence of event duration on automatic ARS classification, namely, how the creation of the *Other* class (negative class) affected the classifiers’ performance. (2) Methods: We conducted a set of experiments where we varied the durations of the other events on three tasks: crackle vs. wheeze vs. other (*3 Class*); crackle vs. other (*2 Class Crackles*); and wheeze vs. other (*2 Class Wheezes*). Four classifiers (linear discriminant analysis, support vector machines, boosted trees, and convolutional neural networks) were evaluated on those tasks using an open access respiratory sound database. (3) Results: While on the *3 Class* task with fixed durations, the best classifier achieved an accuracy of 96.9%, the same classifier reached an accuracy of 81.8% on the more realistic *3 Class* task with variable durations. (4) Conclusion: These results demonstrate the importance of experimental design on the assessment of the performance of automatic ARS classification algorithms. Furthermore, they also indicate, unlike what is stated in the literature, that the automatic classification of ARS is not a solved problem, as the algorithms’ performance decreases substantially under complex evaluation scenarios.

## 1. Introduction

Respiratory diseases are among the most significant causes of morbidity and mortality worldwide [1] and are responsible for a substantial strain on health systems [2]. Early diagnosis and routine monitoring of patients with respiratory conditions are crucial for timely interventions [3]. Health professionals are trained to listen to and to recognize respiratory pathological findings, such as the presence of adventitious respiratory sounds (ARS) (e.g., crackles and wheezes), commonly in the anterior and posterior chest of the patient [4].

Respiratory sounds have been validated as an objective, simple, and noninvasive marker to check the respiratory system [5]. In clinical practice they are commonly assessed with pulmonary auscultation using a stethoscope. Despite the technological advances in auscultation devices, which have enabled the storing, analysis, and visualization of respiratory sounds in computers, digital auscultation is not yet entirely computational. Conventional auscultation is usually employed but has some drawbacks that limit its expansion in clinical practice and suitability in research due to: (i) the necessity of an expert to annotate the presence/absence and clinical meanings of normal/abnormal respiratory sounds [6]; (ii) the unfeasibility of providing continuous monitoring; (iii) its inherent inter-listener variability [7]; (iv) human audition and memory limitations [8]; and (v) as demonstrated during the COVID-19 crisis, it might not be viable in highly contagious situations, as stethoscopes can be a source of infection and need to be constantly sanitized [9]. These limitations could potentially be surmounted by automated respiratory sound analysis.

Respiratory sounds can be normal or abnormal. Normal respiratory sounds are nonmusical sounds provided by breathing and heard over the trachea and chest wall [10]. They show different acoustic properties, such as duration, pitch, and sound quality depending on the characteristics and position of subjects, respiratory flow, and recording location [6,11]. On the other hand, ARS are abnormal sounds that are overlayed on normal respiratory sounds [10]. ARS can be categorized into two main types: continuous and discontinuous [12]. The nomenclature recognized by the European Respiratory Society Task Force on Respiratory Sounds [13] is that continuous ARS are called wheezes, and discontinuous ARS are called crackles, which will be followed in this study.

Crackles are explosive, short, discontinuous, and nonmusical ARS that are attributed to the sudden opening and closing of abnormally closed airways [14]. They usually last less than 20 ms and can be classified as fine or coarse depending on their duration and frequency. Fine crackles have short duration and high frequency, whereas coarse crackles have longer duration and lower frequency [15]. Although the frequency range of crackles is bounded by 60 Hz and 2 kHz, most of their energy is concentrated between 60 Hz and 1.2 kHz [16]. The characteristics of crackles, such as number, regional distribution, timing in the respiratory cycle, and especially the distinction between fine and coarse, can all be used in the diagnosis of various types of lung diseases, such as bronchiectasis or pneumonia [15]. In contrast, wheezes are musical respiratory sounds usually longer than 100 ms. Their typical frequency range is between 100 and 1000 Hz, with harmonics that occasionally exceed 1000 Hz [17]. Wheezes occur when there is flow limitation and can be clinically defined by their duration, intensity, position in the respiratory cycle (inspiratory or expiratory), frequency (monophonic or polyphonic), number, gravity influence, and respiratory maneuvers [14]. Health professionals have utilized wheezes for diagnosing various respiratory conditions in adults (e.g., chronic obstructive pulmonary disease) and in children (e.g., bronchiolitis) [14].

Several authors have reported excellent performance on ARS classification. However, a robust experimental design is lacking in many studies, leading to overestimated results. To determine if a system is relevant, we need to understand the extent to which the characteristics it is extracting from the signal are confounded with the ground truth [18]. In the case of ARS classification, we argue that results in the literature are overestimated because little attention has been dedicated to the design of the negative classes; i.e., the classes against which the wheeze or crackle classification algorithms learn to discriminate.

The main objective of this study was to understand, through a set of experiments with different tasks, how experimental design can impact classification performance. We used four machine learning algorithms in the experiments: linear discriminant analysis (LDA), support vector machines with radial basis function (SVMrbf), random undersampling boosted trees (RUSBoost), and convolutional neural networks (CNNs). The LDA, SVMrbf, and RUSBoost classifiers were fed features extracted from the spectrograms, including some novel acoustic features. On the other hand, the CNNs received spectrogram and mel spectrogram images as inputs.

The article is organized as follows: in Section 2, we provide a general overview of the state-of-the-art on algorithms that have been used in similar works to automatically classify wheezes and crackles; in Section 3, we provide information regarding the dataset, and all the methods used in the different stages of the classification process; in Section 4, the obtained results are presented; and lastly, in Section 5, the results are analyzed and a global conclusion is presented. This paper expands previously published work [19] that focused only on wheeze classification.

## 2. Related Work

Several features and machine learning approaches have been proposed to develop methods for the automatic classification of respiratory sounds [20,21,22,23,24]. In most systems, suitable features are extracted from the signal and are subsequently used to classify ARS (i.e., crackles and wheezes). The most common features and machine learning algorithms employed in the literature to detect or classify ARS have been reported [6], including spectral features [25], mel-frequency cepstral coefficients (MFCCs) [26], entropy [27], wavelet coefficients [28], rule-based models [29], logistic regression models [30], support vector machines (SVM) [31], and artificial neural networks [32]. More recently, deep learning strategies have also been introduced, where the feature extraction and classification steps are merged into the learning algorithm [33,34,35].

Over the years, several authors have reported excellent results on ARS classification (Table 1). However, one crucial problem of this field has been its reliance on small or private data collections. Moreover, public repositories that have been commonly used in the literature (e.g., R.A.L.E. [36]) were designed for teaching, typically including a small number of ARS, and usually not containing environmental noise. Therefore, we chose to perform the evaluation on the Respiratory Sound Dataset (RSD), the largest publicly available respiratory sound database, which is described in Section 3.1.

## 3. Materials and Methods

### 3.1. Database

The ICBHI 2017 Respiratory Sound Database (RSD) is a publicly available database with 920 audio files containing a total of 5.5 h of recordings acquired from 126 participants of all ages [44]. The database (Table 2) contains audio samples collected independently by two research teams in two different countries. It is a challenging database, since the recordings contain several types of noises, background sounds, and different sampling frequencies; 1898 wheezes and 8877 crackles, which are found in 637 audio files, are annotated. The training set contains 1173 wheezes and 5996 crackles distributed among 203 and 311 files, respectively. The test set includes 725 wheezes and 2881 crackles distributed among 138 and 190 files, respectively. Moreover, patient-based splitting was performed following the split suggested by the RSD authors [45].

### 3.2. Random Event Generation

We created a custom script to randomly generate events with fixed durations of 50 ms and 150 ms. This procedure was followed to reproduce “Experiment 2” [44], an experiment where ARS events were classified against other events. By employing this process we were able to establish a fair comparison with other methods that were tested on the same database. To simultaneously guarantee variation and reproducibility, the seed for the random number generator changed for each file but was predetermined. The number of randomly generated events (RGE) of each duration is displayed in Table 3, along with the number of annotated events.

**Figure 1:**
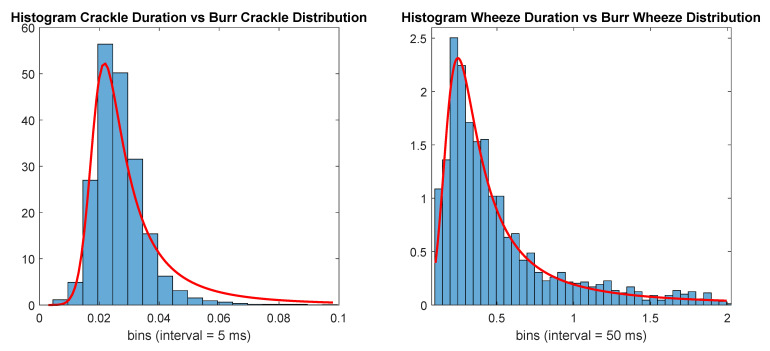
Histogram of adventitious respiratory sounds (ARS) events’ durations versus Burr distributions (red line).

An alternative approach to generate the random events was then employed to study the impacts of event duration on the performance of the classifiers. For this approach, we started by visually inspecting the distribution of the annotated crackles’ and wheezes’ durations and found that a Burr distribution [46] provided a good fit for both distributions. The Burr distribution used to generate the events with durations shorter than 100 ms (otherCrackle) had probability density function

(1) $$f\left(x|a,c,k\right)=\frac{\frac{kc}{\alpha }{\left(\frac{n}{\alpha }\right)}^{c-1}}{{\left(1+{\left(\frac{n}{a}\right)}^{c}\right)}^{k+1}},x>0;\alpha >0;c>0;k>0$$

with α=0.199, c=7.6698, and k=0.3146. Durations longer than 100 ms were discarded. The Burr distribution used to generate the events with durations longer than 100 ms (otherWheeze) had probability density function:

(2) $$f\left(x|a,c,k\right)=\frac{\frac{kc}{\alpha }{\left(\frac{n}{\alpha }\right)}^{c-1}}{{\left(1+{\left(\frac{n}{a}\right)}^{c}\right)}^{k+1}},x>0;\alpha >0;c>0;k>0$$

with α=0.2266, c=4.1906, and k=0.3029. Durations longer than 2 s were discarded. The number of events with durations belonging to each distribution is displayed in Table 4, and the number of annotated events. Figure 1 displays both histograms with the according durations for each class and the Burr distributions used to generate the new random events.

### 3.3. Preprocessing

The audio files in RSD were recorded with different sampling rates. Therefore, we resampled every recording at 4000 Hz, the lowest sampling rate in the database. As the signal of interest was below 2000 Hz, this was considered a good resolution for Fourier analysis.

### 3.4. Time Frequency Representations

To generate the time frequency (TF) images of the audio events, three different representations were used: spectrogram, mel spectrogram, and scalogram. All images obtained with the different methods were normalized between 0 and 1. Moreover, TF representations were computed using MATLAB 2020a. We present only the descriptions and results for the two best performing TF representations, which were the spectrogram and the mel spectrogram.

The spectrogram obtained using the short-time Fourier transform (STFT) is one of the most used tools in audio analysis and processing, since it describes the evolution of the frequency components over time. The STFT representation (F) of a given discrete signal is given by [35]:

(3) $$F\left(n,\omega \right)=\sum_{i=-\infty }^{\infty }i\omega \left(n-i\right)e^{-j\omega }$$

where ω(i) is a window function centered at instant *n*.

The mel scale [47] is a perceptual scale of equally spaced pitches, aiming to match the human perception of sound. The conversion from Hz into mels is performed using Equation (4):

(4) $$m=2595\cdot log_{10}\left(1+\frac{f}{700}\right)$$

**Figure 2:**
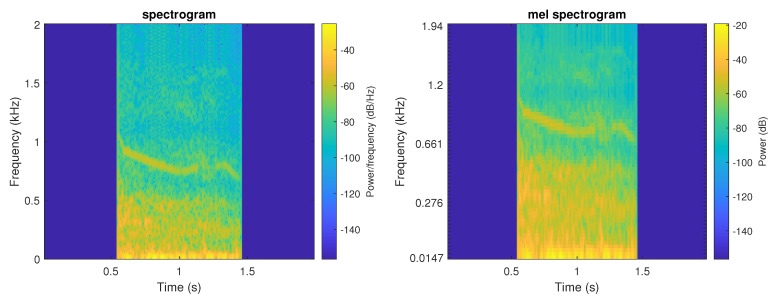
Example of both TF representations of a wheeze event (**left**—spectrogram, **right**—mel spectrogram).

The mel spectogram displays the spectrum of a sound on the mel scale. Figure 2 presents an example of both TF representations.

Since the database events have a wide range of durations, a maximum time for each event was defined according to Equation (5):

(5) Median(x)+2×Std(x),

with *x* corresponding to the durations of annotated wheeze events. Thus, the maximum length per event was established as 2 s, and smaller events were centered and zero-padded. The database also contained annotated events with more than 2 s (87 events). For these cases, only the first 2 s were considered, as we observed that the annotation of these longer events was less precise.

The TF representations were obtained with three windowing methods and three different window lengths: Hamming, Blackman–Harris, and rectangular windows with the respective sizes of 32, 64 ms and 128 ms. We decided to only report the results for the best performing windowing method and window length, the Blackman–Harris window with a size of 32 ms. Moreover, 512 points with 75% overlap were employed to compute the STFT and obtain both TF representations. For the mel spectrogram, 64 mel bandpass filters were employed. The resulting spectrogram and mel spectrogram images were 1 × 247 × 257 and 1 × 247 × 64.

### 3.5. Feature Extraction

To study how frame lengths influence spectrogram computation, a multiscale approach was followed for feature extraction. We computed spectrograms with three windowing methods and six window lengths: Hamming, Blackman–Harris, and rectangular windows with window lengths of 16, 32, 64, 128, 256, and 512 ms with 75% overlap. Then, 81 features were extracted from each frame of the spectrogram: 25 spectral features, 26 MFCC features, and 30 melodic features. Sensitivity analysis on the most realistic task, the *3 Class* task with variable durations, revealed that the Hamming window produced slightly better results. Therefore, all the results obtained with the traditional approach of feature extraction, feature selection, and classification, were computed using the Hamming window. Most features were extracted using the MIR Toolbox 1.7.2 [48]. Table 5 provides a small description of all the employed features. For each event, five statistics of each feature were calculated: mean, standard deviation, median, minimum value, and maximum value. Therefore, the total number of features fed to the classifiers was 2430.

#### 3.5.1. Spectral Features

We estimated several features from the spectrograms. To begin with, the first four standardized moments of the spectral distributions were computed: centroid, spread, skewness, and kurtosis. Then, we extracted other features that are commonly employed for characterizing the timbre of a sound, such as zero-crossing rate, entropy, flatness, roughness, and irregularity. The spectral flux (SF), which measures the Euclidean distance between the magnitude spectrum of successive frames, gave origin to three other features: SF inc, where only positive differences between frames were summed; SF halfwave, a halfwave rectification of the SF; SF median, where a median filter was used to remove spurious peaks. Finally, the amount of high-frequency energy was estimated in two ways: brightness, the high-frequency energy above a certain cut-off frequency; rolloff, which consists of finding the frequency below which a defined percentage of the total spectral energy is contained [48]. Brightness was computed at four frequencies: 100, 200, 400, and 800 Hz. Furthermore, we calculated the ratios between the brightnesses at 400 and 100 Hz, and between the brightnesses at 800 and 100 Hz. Rolloff was computed for the percentages of 95, 75, 25, and 5. Moreover, two novel features were computed: the outlier ratio between rolloffs at 5 and 95%; the interquartile ratio between rolloffs at 25 and 75%.

#### 3.5.2. MFCC Features

The most common features used to describe the spectral shape of a sound are the MFCCs [49]. The MFCCs are calculated by converting the logarithm of the magnitude spectrum to the mel scale and computing the discrete cosine transform (DCT). As most of the signal information is concentrated in the first components, it is typical to extract the first 13 [48]. A first-order temporal differentiation of the MFCCs was also computed to understand the temporal evolution of the coefficients.

#### 3.5.3. Melodic Features

Fundamental frequency, henceforth referred to as pitch, was the basis for computing the 30 melodic features. We computed the cepstral autocorrelation of each frame to estimate each event’s pitch curve. The maximum allowed pitch frequency was 1600 Hz, the highest fundamental frequency reported in the literature about wheezes [50]. The inharmonicity and the voicing curves were then computed based on the pitch curve. Next, we applied moving averages with durations 100, 250, 500, and 1000 ms to the time series to understand trends at different lengths and smooth the curves, giving origin to a total of 15 features. Finally, the same features were computed for a 400 Hz high-pass filtered version of the sound events. The rationale for this filter was the removal of the respiratory sounds, whose energy typically drops at 200 Hz [17], reaching insignificant levels at 400 Hz [50].

### 3.6. Feature Selection

After preliminary experiments, the minimum redundancy maximum relevance (MRMR) algorithm was chosen to perform feature selection. This algorithm provides ranks to the features that are mutually and maximally dissimilar and can represent the response variable effectively [51] The MRMR algorithm ranks features by calculating the mutual information quotient of the relevance and redundancy of each feature. For each experiment, three subsets of features were selected: the best 10 features selected by MRMR (10MRMR), the best 100 features selected by MRMR (100MRMR), and all 2430 features.

Table 6 and Table 7 list the 10 most relevant features as ranked by the MRMR algorithm on both fixed durations (FD) and variable durations (VD) sets. The first noteworthy fact is that, while features from every frame length were selected for all the tasks in the VD set, features extracted with the longest window size (512 ms) were not selected for any task in the FD set. Comparing the feature sets selected for the *3 Class* tasks, while the best 2 features on the FD set were melodic features, the best 2 features and 3 of the best 10 features for the variable durations dataset were spectral. In both cases, 7 MFCC features were present in the 10 highest-ranked features. The novel brightness ratios turned out to be important features, as they were selected for every task in both sets. In the VD set, while no melodic features were selected for the *3 Class* and *2 Class Crackles* tasks, two of the smoothed inharmonicities we introduced were selected for the *2 Class Wheezes* task.

### 3.7. Classifiers

We used four machine learning algorithms to classify the events: linear discriminant analysis (LDA), SVM with radial basis function (SVMrbf), random undersampling boosted trees (RUSBoost), and convolutional neural networks (CNNs). All the classifiers were trained 10 times with different seeds, and their hyperparameters were optimized on a validation set containing 25% of the training set. The models with the best hyperparameters were then applied to the test set. Bayesian optimization [52] was used to optimize the following hyperparameters of each traditional machine learning algorithm: delta for LDA; box constraint and kernel scale for SVMrbf; learning rate, number of variables to sample, number of learning cycles, minimum leaf size, and maximum number of splits for RUSBoost.

**Figure 3:**
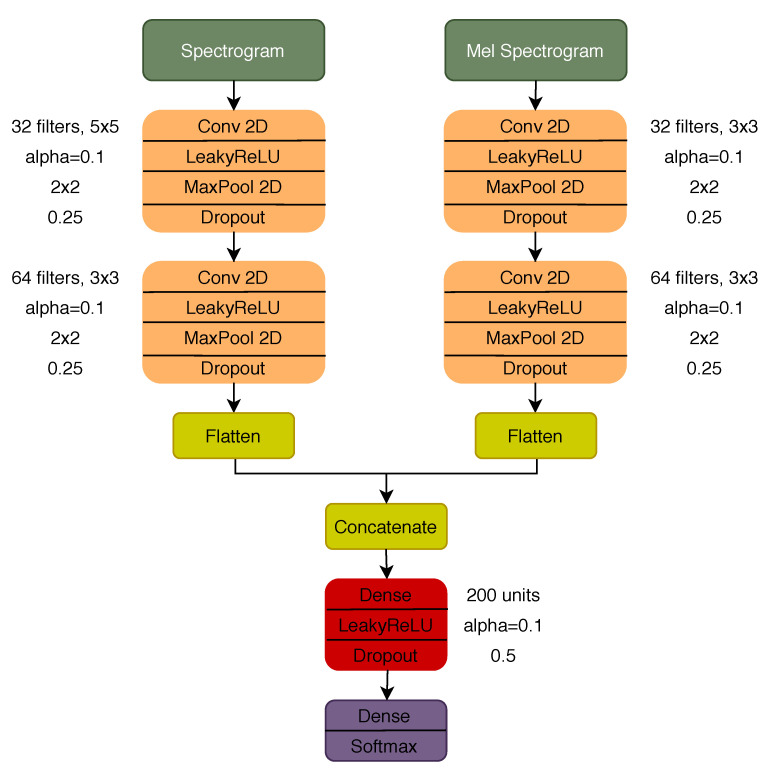
Dual input CNN architecture.

Three different CNN models were considered with regard to deep learning approaches: a model with a dual input configuration, using the spectrogram and mel spectrogram as inputs, and two other models using each of the TF representations individually as input. The architecture of the dual input model and the parameter for each of the layers is represented in Figure 3. The architecture of the models with a single input is the same as the one represented in Figure 3, considering the respective branch before the concatenation and the remaining layer afterwards. To train all the deep learning models, a total of 30 epochs were used with a batch size of 16 and 0.001 learning rate (Adam optimization algorithm). The early stopping strategy [53] was used to avoid overfitting during the training phase, i.e., stopping the training process after 10 consecutive epochs with an increase in the validation loss (validated in 25% of the training set).

### 3.8. Evaluation Metrics

We used the following measures to evaluate the performance of the algorithms:

(6) $$Accuracy=\frac{\left(TP+TN\right)}{\left(TP+TN+FP+FN\right)}$$

(7) $$Precision=\frac{TP}{\left(TP+FP\right)}$$

(8) $$Sensitivity=\frac{TP}{\left(TP+FN\right)}$$

(9) $$F1Score\left(F1\right)=\frac{\left(2\times Precision\times Sensitivity\right)}{\left(Precision+Sensitivity\right)}$$

(10) $$MatthewsCorrelationCoefficient\left(MCC\right)=\frac{\left((TP\times TN)-(FP\times FN)\right)}{\sqrt{\left((TP+FP)(TP+FN)(TN+FP)(TN+FN)\right)}}$$

where TP (True Positives) are events of the relevant class that are correctly classified; TN (True Negatives) are events of the other classes that are correctly classified; FP (False Positives) are events that are incorrectly classified as the relevant class; FN (False Negatives) are events of the relevant class that are incorrectly classified. The area under the ROC curve (AUC) was also computed for the binary cases. For multi-class classification, the evaluation metrics were computed in a one-vs-all fashion. Precision and sensitivity were not included in the tables of Section 4 to improve legibility.

## 4. Evaluation

In this section, we analyze the performance of the algorithms in three experiments that are detailed in the following subsections. Each experiment is composed of three tasks: one problem with three classes, i.e., crackles, wheezes, and others (*3 Class*); and two problems with two classes, i.e., crackles and others (*2 Class Crackles*), and wheezes and others (*2 Class Wheezes*). Each experiment is divided into three tasks in order to study how the performance of the algorithms are affected by having to classify each type of ARS against events of the same range of durations. By partitioning the RGE into two sets, we can determine whether the performance in the *3 Class* problem is inflated.

### 4.1. Fixed Durations

Table 8 displays the results achieved by all the combinations of classifiers and feature sets on the test set of the *3 Class* task with fixed durations. Results achieved by the best performing algorithm in "Experiment 2" of [44], SUK [41], are also shown as a baseline for comparison. Table 9 displays the results achieved by all the combinations of classifiers and feature sets on the test set of the *2 Class Crackles* task with fixed durations. Table 10 displays the results achieved by all the combinations of classifiers and feature sets on the test set of the *2 Class Wheezes* task with fixed durations.

With an accuracy of 95.8%, SVMrbf_MFCC was the best traditional classifier in the *3 Class* task, surpassing the baseline accuracy of 91.2%. Nevertheless, the CNNs achieved even better results, with several reaching 96.9% accuracy. Given such great results, we decided to investigate whether the performance would be the same for two-class tasks, i.e., wheezes vs. 150 ms RGE, and crackles vs. 50 ms RGE. Surprisingly, while the traditional classifiers’ performance did not improve, the CNNs achieved better results in both tasks, with CNN_dualInput reaching 99.6% accuracy and 99.6% AUC in the *2 Class Crackles* task, and 98.6% accuracy and 98.4% AUC in the *2 Class Wheezes* task.

### 4.2. Fixed and Variable Durations

After noticing the CNNs had achieved almost perfect performance on the fixed durations experiment, we suspected the algorithms might be implicitly learning the duration of each event instead of the underlying characteristics of each type of sound. To test this, we designed a new experiment with a different approach to random event generation, detailed in Section 3.2. In this experiment, the training set was the same as before—i.e., the RGE had fixed durations—but the test set’s RGE had variable durations. Table 11 displays the results achieved by all the combinations of classifiers and feature sets on the test set of the *3 Class* task with variable durations. As a baseline, we computed SUK’s results on this test set with the same training model as before. Table 12 displays the results achieved by all the combinations of classifiers and feature sets on the test set of the *2 Class Crackles* task with variable durations. Table 13 displays the results achieved by all the combinations of classifiers and feature sets on the test set of the *2 Class Wheezes* task with variable durations.

Looking at the results of the *3 Class* task, the decline in performance is quite salient, with the accuracy decreasing by more than 30% for the best classifiers. The bulk of this decline was due to the class other, as can be seen in the last three columns of Table 11. With this experiment, we were able to grasp that classifiers were implicitly learning the duration of the events, rather than relevant characteristics of the classes. The performance did not improve in the *2 Class* tasks. In the *2 Class Crackles* task, the highest AUC, reached by SVMrbf_100MRMR, was 68.4%, whereas the AUC attained by the CNNs was close to 50%, thereby not being better than random. In the *2 Class Wheezes* task, the best AUC, reached by SVMrbf_Full, was 57.2%, also close to random.

### 4.3. Variable Durations

Finally, in this experiment we examined whether the algorithms’ performance improved when training with RGE with variable durations. This experiment arguably represents the more realistic setup to evaluate the performance of the classifiers, as we aimed to remove the bias introduced by the generation of random events with fixed sizes. Table 14 displays the results achieved by all the combinations of classifiers and feature sets on the test set of the *3 Class* task with variable durations. Table 15 displays the results achieved by all the combinations of classifiers and feature sets on the test set of the *2 Class Crackles* task with variable durations. Table 16 displays the results achieved by all the combinations of classifiers and feature sets on the test set of the *2 Class Wheezes* task with variable durations.

**Figure 4:**
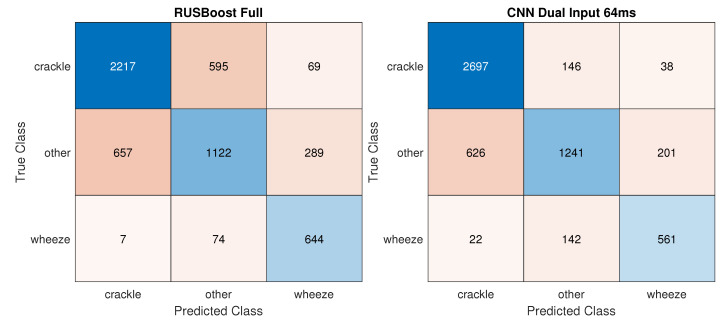
Confusion matrices for the best traditional and deep learning models on the *3 Class* task––training: variable duration; testing: variable duration.

While the accuracy reached by the best traditional classifier RUSBoost_Full increased by 6.2% in the *3 Class* task, the improvement in performance was especially appreciable in the CNNs, with CNN_dualInput reaching 81.8% accuracy an 20.3% increase in accuracy. Figure 4 displays confusion matrices for the best traditional and deep learning models. In the *2 Class Crackles* task, CNN_dualInput achieved the best AUC, 84.9%, not much higher than the best AUC reached by a traditional classifier, SVMrbf_100MRMR, 80.1%. In the two-class wheezes task, traditional and deep learning classifiers attained similar results, 68.5% (SVMrbf_Full) and 72.7% (CNN_dualInput), respectively.

## 5. Discussion

In this work, we proposed a set of experiments that can be used to evaluate ARS classification systems. We demonstrated how random event generation can have a significant impact on the automatic classification of ARS through the evaluation of several classifiers on those experiments. As the performance of the algorithms presented in Section 4 shows, methods that seem to achieve promising results can fail if we change the way the other class is designed. This can happen even if the dataset where the systems are evaluated does not change. The substantial variance in performance between experiments might indicate that the generation of the random events with fixed durations introduces a considerable bias. Classifiers might be implicitly learning to identify the durations of the events. It is important to consider how data are used to train, validate, and test a trained model. Such a model should encode some essential structure of the underlying problem [54]. When a highly specified artificial system appears to give credence to the allegation that it is addressing a complex human task, the default position should be that the system relies upon characteristics confounded with the ground truth and is not actually addressing the problem it appears to be solving [18]. Our findings corroborate the need to test models on realistic and application-specific tasks [54].

Nevertheless, it is important to reiterate that the performance of the evaluated systems may have been influenced by the limitations of this dataset. As previously pointed out [44], these include the shortage of healthy adult participants and the unavailability of gold standard annotations, (i.e., annotations from multiple annotators). A future update of the database should also check for possible errors.

Automatic classification of ARS is a complex task that is not yet solved, despite the claims made in the literature. It may be particularly hard when algorithms are evaluated on challenging datasets, such as the RSD. Though significant work has been developed to classify ARS, none has been widely accepted [55]. While CNNs have become state-of-the-art solutions in several tasks [34], they were not enough to tackle this problem. Therefore, accelerating the development of machine learning algorithms is critical to the future of respiratory sounds analysis. Future work on ARS classification should focus on improving three crucial steps of the methodology: (i) TF representations; (ii) deep learning architectures; and (iii) evaluation. Other TF representations have been proposed for ARS classification, such as the wavelet transform [28], the S-transform [43], and the scalogram [56], but better denoising methods would allow us to extract more meaningful features. Hybrid deep learning architectures that combine convolutional layers with recurrent layers that learn the temporal context have been shown to perform well in other sound event classification tasks [57] and could be successfully applied in ARS classification. Finally, ARS classification systems should be evaluated on realistic datasets containing several noise sources.

## Figures and Tables

**Table 1:** Summary of selected works.

Reference	Data	#Classes: Classes	Best Results
Forkheim et al. [37]	*Participants*: NA; *Recordings*: NA; *Source*: Private	2: Wheezes and Normal	*Accuracy*: 96%
Riella et al. [38]	*Participants*: NA; *Recordings*: 28 *Source* R.A.L.E.	2: Wheezes and Normal	*Accuracy*: 85%; *Sensitivity*: 86%; *Specificity*: 82%
Mendes et al. [39]	*Participants*: 12; *Recordings*: 24; *Source* Private	2: Wheezes and Normal	*Accuracy*: 98%; *Sensitivity*: 91%; *Specificity*: 99%; MCC: 93%
Pinho et al. [29]	*Participants*: 10; *Recordings*: 24; *Source*: Private	1: Crackles	*Precision*: 95%; *Sensitivity*: 89%; *F1*: 92%
Chamberlain et al. [32]	*Participants*: 284; *Recordings*: 500; *Source*: Private	3: Wheezes, Crackles, and Normal	Wheeze *AUC*: 86%; Crackle *AUC*: 73%
Lozano et al. [31]	*Participants*: 30; *Recordings*: 870; *Source*: Private	2: Wheezes and Normal	*Accuracy*: 94%; *Precision*: 95%; *Sensitivity*: 94%; *Specificity*: 94%
Gronnesby et al. [40]	*Participants*: NA; *Recordings*: 383; *Source*: Private	2: Crackles and Normal	*Precision*: 86% *Sensitivity*: 84% *F1*: 84%
Aykanat et al. [33]	*Participants*: 1630; *Recordings*: 17930; *Source*: Private	2: Healthy and Pathological	*Accuracy*: 86%; *Precision*: 86%; *Sensitivity*: 86%; *Specificity*: 86%
Bardou et al. [34]	*Participants*: 15; *Recordings*: 15; *Source*: R.A.L.E.	7, including Wheezes, Crackles, and Normal	*Accuracy*: 96%; Wheeze *Precision*: 98%; Wheeze *Sensitivity*: 100%
Serbes et al. [41]	*Participants*: 126; *Recordings*: 920; *Source*: RSD	3: Wheezes, Crackles, and Normal	Wheeze *Sensitivity*: 79%; Crackle *Sensitivity*: 95%; Normal *Sensitivity*: 91%
Jakovljevic et al. [42]	*Participants*: 126; *Recordings*: 920; *Source*: RSD	3: Wheezes, Crackles, and Normal	Wheeze *Sensitivity*: 52%; Crackle *Sensitivity*: 56%; Normal *Sensitivity*: 52%
Chen et al. [43]	*Participants*: NA; *Recordings*: 240; *Source*: R.A.L.E. and RSD	2: Wheezes and Normal	*Accuracy*: 99%; *Sensitivity*: 96%; *Specificity*: 99%

AUC: area under the receiver operating characteristic curve; F1: F1-score; MCC: the Matthews correlation coefficient; NA: not available; RSD: Respiratory Sound Database.

**Table 2:** Demographic information of the database.

Number of recordings	920
Sampling frequency (number of recordings)	4 kHz (90); 10 kHz (6); 44.1 kHz (824)
Bits per sample	16
Average recording duration	21.5 s
Number of participants	126: 77 adults, 49 children
Diagnosis	COPD (64); Healthy (26); URTI (14); Bronchiectasis (7); Bronchiolitis (6); Pneumonia (6); LRTI (2); Asthma (1)
Sex	79 males, 46 females (NA: 1)
Age (mean ± standard deviation)	43.0 ± 32.2 years (NA: 1)
Age of adult participants	67.6 ± 11.6 years (NA: 1)
Age of child participants	4.8 ± 4.6 years
BMI of adult participants	27.2 ± 5.4 kg m^2^ (NA: 2)
Weight of child participants	21.4 ±17.2 kg (NA: 5)
Height of child participants	104.7 ± 30.8 cm (NA: 7)

COPD: chronic obstructive pulmonary disease; LRTI: lower respiratory tract infection; NA: not available; URTI: upper respiratory tract infection.

**Table 3:** Number of randomly generated events (RGE) with fixed durations in the training and test sets.

	Training Set	Test Set	Total
Number of crackles	5996	2881	8877
Number of wheezes	1173	725	1898
Number of 50 ms events	1557	1050	2607
Number of 150 ms events	1456	962	2418

**Table 4:** Number of RGE with variable durations in the training and test sets.

	Training Set	Test Set	Total
Number of crackles	5996	2881	8877
Number of wheezes	1173	725	1898
Number of otherCrackle events	2478	1680	4158
Number of otherWheeze events	575	388	963

**Table 5:** Small description of each feature.

Type	Features	Description
Spectral	Spectral Centroid	Center of mass of the spectral distribution
	Spectral Spread	Variance of the spectral distribution
	Spectral Skewness	Skewness of the spectral distribution
	Spectral Kurtosis	Excess kurtosis of the spectral distribution
	Zero-crossing Rate	Waveform sign-change rate
	Spectral Entropy	Estimation of the complexity of the spectrum
	Spectral Flatness	Estimation of the noisiness of a spectrum
	Spectral Roughness	Estimation of the sensory dissonance
	Spectral Irregularity	Estimation of the spectral peaks’ variability
	Spectral Flux	Euclidean distance between the spectrum of successive frames
	Spectral Flux Inc	Spectral flux with focus on increasing energy solely
	Spectral Flux Halfwave	Halfwave rectified spectral flux
	Spectral Flux Median	Median filtered spectral flux
	Spectral Brightness	Amount of energy above 100, 200, 400, and 800 Hz
	Brightness 400 Ratio	Ratio between spectral brightness at 400 and 100 Hz
	Brightness 800 Ratio	Ratio between spectral brightness at 800 and 100 Hz
	Spectral Rolloff	Frequency such that 95, 75, 25, and 5% of the total energy is contained below it
	Rolloff Outlier Ratio	Ratio between spectral rolloff at 5 and 95%
	Rolloff Interquartile Ratio	Ratio between spectral rolloff at 25 and 75%
MFCC	MFCC	13 Mel-frequency cepstral coefficients
	Delta-MFCC	1st-order temporal differentiation of the MFCCs
Melodic	Pitch	Fundamental frequency estimation
	Pitch Smoothing	Moving average of the pitch curve with lengths of 100, 250, 500, and 1000 ms
	Inharmonicity	Partials non-multiple of fundamental frequency
	Inharmonicity Smoothing	Moving average of the inharmonicity curve with lengths of 100, 250, 500, and 1000 ms
	Voicing	Presence of fundamental frequency
	Voicing Smoothing	Moving average of the voicing curve with lengths of 100, 250, 500, and 1000 ms

**Table 6:** Ten highest-ranked features (fixed durations).

Rank	3 Class	2 Class Crackles	2 Class Wheezes
1	std_melinharm250ms_32	std_melinharm250ms_32	std_melinharm500ms_64
2	median_melvoicing_16	max_melinharm_64	median_melpitchHF_16
3	std_deltamfcc2_64	min_specbright4ratio_32	std_melpitchHF_128
4	std_deltamfcc10_64	max_speccentroid_256	std_specrolloff05_32
5	median_specbright4ratio_32	min_mfcc11_16	std_specrolloff05_128
6	median_deltamfcc7_32	min_deltamfcc11_32	max_melvoicingHF_128
7	min_deltamfcc5_128	std_deltamfcc3_32	std_specbright4ratio_256
8	median_deltamfcc13_32	median_deltamfcc13_16	mean_melinharmHF250ms_32
9	max_mfcc2_64	min_deltamfcc5_32	std_specrolloff05_16
10	median_deltamfcc1_32	min_deltamfcc7_16	max_mfcc12_256

min: minimum; max: maximum; std: standard deviation; spec: spectral; mel: melodic; inharm: inharmonicity; HF: high-frequency; rolloffOutRatio: rolloff outlier ratio; rolloffIQRatio: rolloff interquartile ratio; bright8ratio: brightness 800 ratio; bright4ratio: brightness 400 ratio.

**Table 7:** Ten highest-ranked features (variable durations).

Rank	3 Class	2 Class Crackles	2 Class Wheezes
1	std_specentropy_128	min_specbright4ratio_32	mean_specbright8ratio_16
2	std_specskewness_64	max_speccentroid_128	std_mfcc5_512
3	min_deltamfcc12_64	min_deltamfcc7_32	std_melinharm250ms_16
4	std_specbright8ratio_64	min_deltamfcc3_16	mean_mfcc11_32
5	mean_deltamfcc13_512	median_deltamfcc6_32	mean_deltamfcc1_64
6	median_deltamfcc1_32	mean_deltamfcc13_64	std_mfcc5_128
7	max_mfcc11_256	max_mfcc11_64	std_melinharmHF1s_16
8	min_deltamfcc10_256	mean_specirregularity_512	min_deltamfcc5_512
9	median_deltamfcc10_32	max_deltamfcc1_256	std_deltamfcc3_32
10	std_mfcc5_16	max_deltamfcc8_128	median_deltamfcc5_16

min: minimum; max: maximum; std: standard deviation; spec: spectral; mel: melodic; inharm: inharmonicity; HF: high-frequency; rolloffOutRatio: rolloff outlier ratio; rolloffIQRatio: rolloff interquartile ratio; bright8ratio: brightness 800 ratio; bright4ratio: brightness 400 ratio.

**Table 8:** Performance results obtained with 3 classes (crackle vs. wheeze vs. other)—training: fixed duration; testing: fixed duration.

Classifiers	Accuracy	F1Wheez	MCCWheez	F1Crack	MCCCrack	F1Other	MCCOther
SUK (Baseline)	91.2	77.8	74.5	95.1	90.0	90.5	85.2
LDA_10MRMR	80.4 ± 0.0	41.0 ± 0.1	35.2 ± 0.1	92.4 ± 0.0	85.4 ± 0.0	76.4 ± 0.0	61.9 ± 0.1
LDA_100MRMR	81.1 ± 0.7	63.1 ± 0.9	58.5 ± 0.7	91.8 ± 0.0	85.5 ± 0.0	75.5 ± 1.4	61.8 ± 1.7
LDA_Full	84.2 ± 1.4	70.9 ± 1.5	66.6 ± 1.7	91.0 ± 0.6	81.7 ± 1.7	79.5 ± 2.6	69.0 ± 3.5
SVMrbf_10MRMR	82.9 ± 0.3	61.0 ± 2.9	55.6 ± 3.2	91.3 ± 0.5	82.3 ± 1.1	78.5 ± 0.5	66.1 ± 0.9
SVMrbf_100MRMR	88.3 ± 0.3	76.9 ± 0.5	73.8 ± 0.6	92.5 ± 0.3	84.7 ± 0.7	86.2 ± 0.3	78.4 ± 0.5
SVMrbf_Full	89.7 ± 1.0	76.8 ± 3.0	74.1 ± 3.1	93.9 ± 0.5	87.8 ± 0.9	88.1 ± 1.2	81.2 ± 2.0
RUSBoost_10MRMR	89.7 ± 0.4	82.4 ± 1.1	79.9 ± 1.5	92.6 ± 0.4	85.2 ± 0.9	88.4 ± 0.5	82.0 ± 0.7
RUSBoost_100MRMR	91.3 ± 0.5	83.7 ± 1.0	81.3 ± 1.2	93.9 ± 0.4	87.8 ± 0.9	90.5 ± 0.6	85.1 ± 1.0
RUSBoost_Full	92.3 ± 1.3	84.9 ± 1.9	82.7 ± 2.2	94.6 ± 0.8	89.2 ± 1.3	91.7 ± 1.7	87.0 ± 2.7
CNN_dualInput	96.9 ± 0.3	89.3 ± 0.9	87.7 ± 1.0	97.7 ± 0.2	95.3 ± 0.4	98.4 ± 0.6	97.6 ± 0.9
CNN_Spectrogram	96.2 ± 0.3	88.1 ± 0.8	86.4 ± 0.8	96.8 ± 0.3	93.4 ± 0.6	98.2 ± 0.3	97.3 ± 0.4
CNN_melSpectrogram	96.7 ± 0.2	88.9 ± 0.9	87.3 ± 1.0	97.5 ± 0.2	94.8 ± 0.5	98.4 ± 0.3	97.6 ± 0.4

**Table 9:** Performance results obtained with 2 classes (crackle vs. other)—training: fixed duration; testing: fixed duration.

Classifiers	Accuracy	AUCCrack	F1Crack	MCCCrack	F1Other	MCCOther
LDA_10MRMR	88.9 ± 0.6	93.9 ± 0.7	92.0 ± 0.3	85.1 ± 1.2	81.9 ± 1.9	78.3 ± 2.7
LDA_100MRMR	88.9 ± 0.0	92.8 ± 0.6	91.8 ± 0.0	85.5 ± 0.0	82.8 ± 0.0	79.9 ± 0.0
LDA_Full	88.1 ± 0.2	93.0 ± 0.2	91.5 ± 0.2	83.6 ± 0.3	79.9 ± 0.4	75.3 ± 0.4
SVMrbf_10MRMR	91.2 ± 0.2	95.2 ± 0.9	94.0 ± 0.1	87.7 ± 0.3	83.5 ± 0.5	79.7 ± 0.6
SVMrbf_100MRMR	93.3 ± 0.2	97.6 ± 0.4	95.4 ± 0.1	90.6 ± 0.2	87.4 ± 0.3	84.5 ± 0.4
SVMrbf_Full	93.5 ± 0.6	97.7 ± 0.3	95.6 ± 0.4	91.0 ± 0.9	88.0 ± 1.2	85.3 ± 1.5
RUSBoost_10MRMR	93.1 ± 0.3	97.5 ± 0.1	95.3 ± 0.2	90.4 ± 0.4	87.4 ± 0.7	84.5 ± 0.8
RUSBoost_100MRMR	95.2 ± 0.7	98.8 ± 0.2	96.7 ± 0.5	93.2 ± 1.0	91.0 ± 1.2	88.9 ± 1.4
RUSBoost_Full	94.7 ± 0.9	98.8 ± 0.4	96.3 ± 0.7	92.6 ± 1.2	90.4 ± 1.4	88.3 ± 1.6
CNN_dualInput	99.6 ± 0.1	99.6 ± 0.2	99.8 ± 0.1	99.1 ± 0.3	99.3 ± 0.2	99.1 ± 0.3
CNN_Spectrogram	98.5 ± 0.5	97.8 ± 1.1	99.0 ± 0.3	96.2 ± 1.2	97.2 ± 0.9	96.2 ± 1.2
CNN_melSpectrogram	99.4 ± 0.2	99.2 ± 0.5	99.6 ± 0.2	98.5 ± 0.6	98.9 ± 0.5	98.5 ± 0.6

**Table 10:** Performance results obtained with 2 classes (wheeze vs. other)—training: fixed duration; testing: fixed duration.

Classifiers	Accuracy	AUCWheez	F1Wheez	MCCWheez	F1Other	MCCOther
LDA_10MRMR	82.5 ± 0.2	83.0 ± 1.1	75.2 ± 0.5	74.6 ± 0.4	86.5 ± 0.2	84.1 ± 0.2
LDA_100MRMR	84.1 ± 0.0	88.6 ± 0.0	77.3 ± 0.1	77.2 ± 0.1	87.8 ± 0.1	85.8 ± 0.0
LDA_Full	83.3 ± 0.2	83.6 ± 0.1	78.1 ± 0.2	76.1 ± 0.2	86.5 ± 0.3	83.9 ± 0.5
SVMrbf_10MRMR	84.4 ± 1.3	87.3 ± 1.1	80.3 ± 2.3	78.1 ± 2.2	87.1 ± 0.8	84.4 ± 0.9
SVMrbf_100MRMR	87.2 ± 0.4	92.8 ± 1.4	84.1 ± 0.9	82.2 ± 0.7	89.3 ± 0.4	87.1 ± 0.5
SVMrbf_Full	88.6 ± 0.4	92.5 ± 1.1	86.1 ± 0.5	84.2 ± 0.6	90.3 ± 0.4	88.3 ± 0.4
RUSBoost_10MRMR	91.6 ± 1.2	96.2 ± 0.7	89.9 ± 1.5	88.6 ± 1.7	92.7 ± 1.1	91.3 ± 1.3
RUSBoost_100MRMR	91.0 ± 1.0	96.5 ± 0.6	89.7 ± 1.2	88.2 ± 1.4	91.9 ± 0.9	90.3 ± 1.1
RUSBoost_Full	93.6 ± 2.0	97.8 ± 0.8	92.5 ± 2.2	91.4 ± 2.6	94.4 ± 1.7	93.3 ± 2.1
CNN_dualInput	98.2 ± 0.4	98.1 ± 0.4	97.9 ± 0.5	96.4 ± 0.9	98.5 ± 0.4	96.4 ± 0.9
CNN_Spectrogram	98.6 ± 0.2	98.4 ± 0.2	98.3 ± 0.2	97.1 ± 0.4	98.8 ± 0.2	97.1 ± 0.4
CNN_melSpectrogram	98.3 ± 0.3	98.1 ± 0.2	97.9 ± 0.3	96.4 ± 0.6	98.5 ± 0.2	96.4 ± 0.6

**Table 11:** Performance results obtained with 3 classes (crackle vs. wheeze vs. other)—training: fixed duration; testing: variable duration.

Classifiers	Accuracy	F1Wheez	MCCWheez	F1Crack	MCCCrack	F1Other	MCCOther
SUK (Baseline)	63.3	68.1	63.5	76.8	47.1	21.7	14.6
LDA_10MRMR	60.3 ± 0.1	45.0 ± 0.1	42.4 ± 0.0	75.1 ± 0.1	43.2 ± 0.2	36.3 ± 0.0	9.8 ± 0.1
LDA_100MRMR	61.1 ± 0.0	69.2 ± 0.5	65.0 ± 0.6	73.5 ± 0.0	39.7 ± 0.0	28.8 ± 0.4	9.3 ± 0.1
LDA_Full	62.9 ± 0.3	66.6 ± 0.4	61.7 ± 0.6	76.1 ± 0.4	45.4 ± 1.0	28.6 ± 1.4	13.9 ± 0.6
SVMrbf_10MRMR	61.9 ± 0.1	60.3 ± 2.5	54.9 ± 2.3	75.7 ± 0.2	44.2 ± 0.5	31.5 ± 1.2	12.0 ± 0.3
SVMrbf_100MRMR	63.7 ± 0.2	68.1 ± 0.4	63.3 ± 0.5	76.5 ± 0.1	46.4 ± 0.4	29.1 ± 0.6	15.7 ± 0.5
SVMrbf_Full	63.6 ± 0.5	66.9 ± 2.4	62.0 ± 2.8	77.0 ± 0.2	47.5 ± 0.5	28.6 ± 1.9	14.4 ± 0.8
RUSBoost_10MRMR	62.1 ± 0.5	68.6 ± 1.7	64.8 ± 2.4	75.8 ± 0.1	44.4 ± 0.4	20.5 ± 1.3	10.5 ± 1.6
RUSBoost_100MRMR	62.7 ± 0.2	69.3 ± 0.8	65.5 ± 1.3	76.2 ± 0.2	45.6 ± 0.5	20.3 ± 3.2	11.7 ± 1.0
RUSBoost_Full	62.9 ± 0.4	70.8 ± 0.7	67.2 ± 0.8	76.4 ± 0.6	46.1 ± 1.4	19.0 ± 4.6	11.4 ± 1.8
CNN_dualInput	61.5 ± 0.4	73.0 ± 0.9	69.6 ± 1.2	75.4 ± 0.4	43.6 ± 1.2	3.4 ± 0.5	3.2 ± 0.9
CNN_Spectrogram	61.7 ± 0.4	71.5 ± 0.8	68.0 ± 1.2	75.6 ± 0.4	43.9 ± 1.1	7.5 ± 1.2	7.7 ± 0.9
CNN_melSpectrogram	61.6 ± 0.3	72.0 ± 0.8	68.8 ± 1.0	75.9 ± 0.3	44.8 ± 0.9	4.3 ± 1.0	3.9 ± 1.3

**Table 12:** Performance results obtained with 2 classes (crackle vs. other)—training: fixed duration; testing: variable duration.

Classifiers	Accuracy	AUCCrack	F1Crack	MCCCrack	F1Other	MCCOther
LDA_10MRMR	62.6 ± 0.8	66.4 ± 2.6	74.7 ± 0.8	42.2 ± 1.8	28.7 ± 0.8	15.5 ± 1.2
LDA_100MRMR	61.5 ± 0.0	67.6 ± 0.6	73.5 ± 0.0	39.7 ± 0.0	29.1 ± 0.0	13.8 ± 0.0
LDA_Full	65.7 ± 0.3	70.5 ± 0.0	76.4 ± 0.1	46.9 ± 0.4	37.3 ± 0.8	24.7 ± 0.8
SVMrbf_10MRMR	65.5 ± 0.1	66.0 ± 0.9	77.5 ± 0.1	49.1 ± 0.2	26.5 ± 0.6	20.9 ± 0.5
SVMrbf_100MRMR	66.1 ± 0.1	68.4 ± 2.0	78.1 ± 0.1	50.7 ± 0.1	25.1 ± 0.7	22.3 ± 0.5
SVMrbf_Full	65.7 ± 0.1	56.9 ± 2.1	77.8 ± 0.1	50.0 ± 0.3	24.1 ± 1.2	20.8 ± 0.5
RUSBoost_10MRMR	65.3 ± 0.3	54.5 ± 0.8	77.5 ± 0.1	49.0 ± 0.4	24.4 ± 1.3	19.7 ± 1.0
RUSBoost_100MRMR	64.6 ± 0.3	54.8 ± 0.5	77.6 ± 0.1	49.8 ± 0.3	15.5 ± 2.0	15.7 ± 1.3
RUSBoost_Full	65.1 ± 0.3	55.3 ± 1.3	77.5 ± 0.3	49.1 ± 1.0	22.6 ± 3.8	18.8 ± 1.6
CNN_dualInput	63.6 ± 0.3	50.7 ± 0.4	77.6 ± 0.1	7.5 ± 1.9	3.0 ± 1.7	7.5 ± 1.9
CNN_Spectrogram	64.2 ± 0.2	51.6 ± 0.3	77.8 ± 0.1	11.6 ± 1.5	7.1 ± 1.4	11.6 ± 1.5
CNN_melSpectrogram	63.6 ± 0.1	50.7 ± 0.2	77.6 ± 0.0	7.9 ± 1.0	3.4 ± 0.7	7.9 ± 1.0

**Table 13:** Performance results obtained with 2 classes (wheeze vs. other)—training: fixed duration; testing: variable duration.

Classifiers	Accuracy	AUCWheez	F1Wheez	MCCWheez	F1Other	MCCOther
LDA_10MRMR	53.3 ± 0.4	55.4 ± 0.0	63.6 ± 0.5	58.4 ± 0.5	35.2 ± 0.2	30.3 ± 0.2
LDA_100MRMR	53.7 ± 0.6	56.2 ± 1.5	63.8 ± 0.7	58.7 ± 0.7	35.8 ± 0.2	30.9 ± 0.3
LDA_Full	56.6 ± 0.9	56.8 ± 0.7	67.3 ± 1.0	62.5 ± 1.1	35.2 ± 0.2	30.7 ± 0.3
SVMrbf_10MRMR	57.3 ± 1.4	49.1 ± 2.2	69.6 ± 2.1	65.1 ± 2.5	27.2 ± 4.2	23.5 ± 3.6
SVMrbf_100MRMR	57.4 ± 1.7	53.5 ± 1.4	70.3 ± 1.6	65.9 ± 1.9	24.7 ± 2.6	21.2 ± 2.6
SVMrbf_Full	61.2 ± 0.6	57.2 ± 1.1	73.4 ± 0.5	69.5 ± 0.6	28.9 ± 1.8	26.4 ± 1.7
RUSBoost_10MRMR	61.2 ± 0.9	51.7 ± 0.6	74.8 ± 0.7	71.6 ± 0.9	15.4 ± 2.3	14.8 ± 2.3
RUSBoost_100MRMR	62.4 ± 0.5	53.2 ± 0.5	76.0 ± 0.3	73.3 ± 0.5	12.7 ± 1.7	13.4 ± 1.8
RUSBoost_Full	61.3 ± 0.8	52.7 ± 1.9	75.6 ± 0.7	73.0 ± 1.0	5.8 ± 1.3	5.5 ± 1.1
CNN_dualInput	64.1 ± 0.1	50.2 ± 0.1	77.9 ± 0.1	−1.0 ± 0.7	4.7 ± 0.1	−1.0 ± 0.7
CNN_Spectrogram	64.1 ± 0.0	51.2 ± 0.0	77.9 ± 0.0	−1.2 ± 0.2	4.8 ± 0.0	−1.2 ± 0.2
CNN_melSpectrogram	64.0 ± 0.5	50.2 ± 0.2	77.8 ± 0.5	−1.1 ± 1.0	5.1 ± 1.2	−1.1 ± 1.0

**Table 14:** Performance results obtained with 3 classes (crackle vs. wheeze vs. other)—training: variable duration; testing: variable duration.

Classifiers	Accuracy	F1Wheez	MCCWheez	F1Crack	MCCCrack	F1Other	MCCOther
LDA_10MRMR	62.3 ± 0.1	71.0 ± 0.0	67.8 ± 0.0	75.2 ± 0.1	42.5 ± 0.1	17.1 ± 0.2	14.2 ± 0.3
LDA_100MRMR	65.5 ± 0.0	72.3 ± 0.1	69.8 ± 0.1	76.7 ± 0.1	47.8 ± 0.1	35.0 ± 0.4	22.5 ± 0.1
LDA_Full	68.8 ± 0.1	72.2 ± 0.1	69.9 ± 0.1	78.2 ± 0.2	52.9 ± 0.2	48.9 ± 0.5	32.5 ± 0.3
SVMrbf_10MRMR	65.6 ± 0.4	72.5 ± 0.5	69.1 ± 0.7	76.7 ± 0.3	47.2 ± 0.7	34.9 ± 2.7	23.2 ± 1.3
SVMrbf_100MRMR	68.2 ± 0.9	68.8 ± 1.9	64.1 ± 2.2	77.4 ± 0.7	51.2 ± 1.4	52.1 ± 2.3	31.3 ± 2.2
SVMrbf_Full	68.0 ± 1.1	65.2 ± 4.0	60.9 ± 3.9	75.9 ± 1.6	51.1 ± 1.5	57.7 ± 3.2	33.4 ± 1.7
RUSBoost_10MRMR	65.4 ± 0.4	72.7 ± 0.6	69.9 ± 0.8	74.8 ± 0.9	45.2 ± 0.8	43.2 ± 3.8	24.1 ± 1.6
RUSBoost_100MRMR	68.5 ± 0.5	73.6 ± 0.8	71.0 ± 1.2	75.4 ± 1.3	50.6 ± 1.0	55.2 ± 2.5	33.6 ± 1.1
RUSBoost_Full	69.0 ± 1.1	73.7 ± 0.7	70.7 ± 0.7	75.4 ± 1.6	51.6 ± 1.7	57.7 ± 0.6	35.2 ± 1.6
CNN_dualInput	81.8 ± 0.7	72.5 ± 2.3	69.3 ± 2.0	88.2 ± 0.6	75.2 ± 1.3	75.1 ± 1.3	62.1 ± 1.4
CNN_Spectrogram	78.7 ± 0.9	70.5 ± 3.0	66.3 ± 3.1	86.2 ± 0.6	70.9 ± 1.7	69.6 ± 2.6	55.9 ± 1.8
CNN_melSpectrogram	76.9 ± 1.3	70.3 ± 2.6	66.2 ± 2.4	84.7 ± 0.8	67.4 ± 2.0	66.3 ± 3.9	51.4 ± 3.0

**Table 15:** Performance results obtained with 2 classes (crackle vs. other)—training: variable duration; testing: variable duration.

Classifiers	Accuracy	AUC	F1Crack	MCCCrack	F1Other	MCCOther
LDA_10MRMR	68.1 ± 0.2	74.7 ± 0.0	76.9 ± 0.1	49.4 ± 0.3	48.4 ± 0.5	33.3 ± 0.5
LDA_100MRMR	70.2 ± 0.3	76.3 ± 0.2	76.4 ± 0.2	52.2 ± 0.5	59.7 ± 1.6	42.7 ± 1.5
LDA_Full	68.5 ± 0.7	73.4 ± 1.1	74.9 ± 1.2	49.4 ± 1.1	57.5 ± 2.2	39.6 ± 2.0
SVMrbf_10MRMR	68.7 ± 0.2	72.2 ± 0.5	78.6 ± 0.1	52.2 ± 0.3	41.4 ± 1.0	31.7 ± 0.8
SVMrbf_100MRMR	72.6 ± 0.5	80.1 ± 0.8	78.6 ± 0.9	56.1 ± 0.9	61.8 ± 1.5	46.6 ± 0.9
SVMrbf_Full	71.2 ± 1.3	78.6 ± 1.4	77.2 ± 1.8	53.7 ± 2.0	60.6 ± 1.4	44.4 ± 1.3
RUSBoost_10MRMR	69.6 ± 0.3	76.0 ± 0.5	76.4 ± 0.6	51.2 ± 0.4	56.9 ± 2.3	40.1 ± 1.9
RUSBoost_100MRMR	71.0 ± 0.7	79.7 ± 0.4	76.9 ± 0.8	53.4 ± 1.1	61.0 ± 0.7	44.4 ± 1.0
RUSBoost_Full	69.9 ± 1.3	78.6 ± 0.9	75.0 ± 1.4	52.0 ± 2.0	62.4 ± 1.4	45.1 ± 2.1
CNN_dualInput	87.4 ± 1.4	84.9 ± 2.3	90.5 ± 0.9	73.0 ± 2.7	81.4 ± 3.0	73.0 ± 2.7
CNN_Spectrogram	86.5 ± 1.3	83.8 ± 2.3	89.8 ± 0.7	70.8 ± 2.5	79.9 ± 3.1	70.8 ± 2.5
CNN_melSpectrogram	85.1 ± 1.2	81.8 ± 2.0	88.9 ± 0.7	67.7 ± 2.6	77.4 ± 2.9	67.7 ± 2.6

**Table 16:** Performance results obtained with 2 classes (wheeze vs. other)—training: variable duration; testing: variable duration.

Classifiers	Accuracy	AUCWheez	F1Wheez	MCCWheez	F1Other	MCCOther
LDA_10MRMR	62.4 ± 0.1	62.5 ± 0.1	73.9 ± 0.2	70.2 ± 0.1	32.5 ± 0.1	30.0 ± 0.0
LDA_100MRMR	55.7 ± 0.9	60.1 ± 1.4	62.2 ± 1.6	57.8 ± 1.5	46.2 ± 1.6	42.3 ± 1.8
LDA_Full	56.5 ± 1.9	59.1 ± 1.7	63.8 ± 2.7	59.4 ± 2.7	45.0 ± 2.7	40.9 ± 3.0
SVMrbf_10MRMR	63.4 ± 0.9	63.8 ± 0.4	72.5 ± 1.1	68.4 ± 1.2	45.3 ± 1.0	41.6 ± 1.0
SVMrbf_100MRMR	66.2 ± 0.9	68.4 ± 1.6	74.6 ± 0.9	70.8 ± 1.0	49.2 ± 3.1	45.8 ± 3.0
SVMrbf_Full	65.4 ± 1.2	68.5 ± 0.7	72.0 ± 1.9	68.4 ± 1.9	54.2 ± 2.3	50.8 ± 2.4
RUSBoost_10MRMR	64.1 ± 1.0	67.7 ± 0.7	70.6 ± 1.4	66.8 ± 1.4	53.6 ± 1.8	50.1 ± 2.0
RUSBoost_100MRMR	64.3 ± 1.5	68.2 ± 0.5	71.1 ± 2.5	67.3 ± 2.3	53.2 ± 1.8	49.8 ± 2.0
RUSBoost_Full	60.9 ± 2.5	65.8 ± 1.9	66.8 ± 3.5	63.1 ± 3.3	52.3 ± 1.4	48.9 ± 1.7
CNN_dualInput	73.2 ± 0.7	72.7 ± 1.1	78.4 ± 1.0	44.0 ± 1.6	64.8 ± 1.6	44.0 ± 1.6
CNN_Spectrogram	69.2 ± 1.8	66.6 ± 1.5	76.0 ± 2.8	33.3 ± 2.4	56.5 ± 3.1	33.3 ± 2.4
CNN_melSpectrogram	69.9 ± 1.3	66.7 ± 1.6	76.9 ± 1.6	33.6 ± 2.6	56.4 ± 2.9	33.6 ± 2.6

## Data Availability

The data used in this study are available in a publicly accessible repository: https://bhichallenge.med.auth.gr/ICBHI_2017_Challenge.
